# A follow up study of seasonality in affective disorders: A preliminary study

**DOI:** 10.4103/0019-5545.31518

**Published:** 2007

**Authors:** Ajit Avasthi, Nitasha Khehra, Nitin Gupta

**Affiliations:** Department of Psychiatry, Post Graduate Institute of Medical Education and Research, Chandigarh, India

**Keywords:** Affective disorder, seasonality, seasonal pattern assessment questionnaire

## Abstract

**Background:**

Researchers have evinced interest in the effect of seasonality on certain behavioural and emotional disorders, the most prominent being Affective Disorders.

**Aim:**

To assess the pattern of seasonality and the clinical course in cases of affective disorder in a tertiary care psychiatric centre in North India.

**Materials and Methods:**

Thirteen patients diagnosed as cases of affective disorder as per ICD- 10 DCR (F30-39) were re-assessed using seasonal pattern assessment questionnaire (SPAQ) after a period of 5-7 years.

**Results:**

Majority of the patients (53.8%) were males. Recurrent depressive disorder current episode moderate with/without somatic syndrome was the commonest current diagnosis as per ICD-10 DCR. Mean global seasonality score was 3.46 (S.D, 6.55; range 1-24) as assessed by SPAQ. Most of the patients did not report any variation in mood, behaviour, sleep pattern and weight fluctuations.

**Conclusions:**

It has been demonstrated that manifestation of an affective disorder is not necessarily associated with high seasonality change.

## INTRODUCTION

Seasonal changes in mood and behavior are well documented.[1] There is a general consensus that seasonal changes in mood, behavior and vegetative functions vary in magnitude across the general population and on the extreme end of the spectrum of seasonality exists the phenomenon of seasonal affective disorder (SAD).[2] SAD is a sub-type of affective disorder that causes severe distress but has non-definitive treatment modalities (apart from phototherapy) available till date.

Seasonal pattern assessment questionnaire (SPAQ)[3] is one of the modalities of assessment. The SPAQ is not sensitive enough to be considered a diagnostic instrument for SAD but nevertheless, it is accurate enough to be used as a screening instrument.[4] Given the long history of its use and advantages in terms of its good psychometric properties and simplicity, the continued use of SPAQ as a dimensional measure of seasonality is indicated.[5]

Seasonality in affective disorder has not received due attention from researchers in India. There is some evidence from the Indian sub-continent that the patterns of seasonality and prevalence and type of SAD may be different.[6–10]

As such, prospective studies are rarer of seasonal effects on affective disorder. Most studies are retrospective and have not used standardized instrument for seasonality. The importance of longitudinal (follow-up) studies in such patients in a structured manner has been stressed.[1] There are available a few follow up studies on SAD, the longest with a mean follow up period of 10.4 years.[10–15]

This prompted us to attempt a study of seasonality in a cohort of follow up patients diagnosed as cases of affective disorder and re-assessed after a period of 5-7 years, using a standardized method of assessment i.e., SPAQ that can be useful to pick up the seasonality linked fluctuations and identify people at risk for SAD.

## MATERIALS AND METHODS

The main aim of the present study was to assess the pattern of seasonality (using SPAQ) and the clinical course in a group of 13 follow up patients diagnosed as cases of affective disorder as per ICD-10 DCR[16] (F30-39) as a part of a previous study in 1998.[17] These patients were attending the outpatient service of the Department of Psychiatry, PGIMER, Chandigarh and were re-assessed after a period of 5-7 years of follow up. Presence of any co-morbid psychiatric illness organic brain disease and alcohol and drug abuse served as the exclusion criteria. The written informed consent was obtained from the patients and the relatives after explaining to them the purpose and nature of the study. No new treatment was envisaged nor was any indicated treatment withheld. Thereafter, they were administered and were assessed on the following instruments:

- The Socio demographic profile sheet developed by the Department of Psychiatry, PGIMER, Chandigarh routinely used to assess the patient's age, sex, marital status, occupation, education, religion, family type and locality.
- SPAQ[3] was used to determine patterns of mood and behavior change over different seasons. It also assesses the severity of seasonal change in several items: sleeping, socializing, mood, weight, appetite and energy. Global seasonality score ranges between 0-24. It also determines the pattern of seasonal change and sensitivity to climate and weather changes.
- Clinical Profile sheet: All patients were also assessed on a specifically designed structured proforma to determine the number of episodes, duration of illness, severity in each episode, symptom profile for depression and mania, family history of psychiatric illness, especially affective disorder.

Seasons were defined as follows: In North India plains, the latitude varies from 27 degrees to 29 degrees north. The climatic conditions are harsh, both in winters as well in the summer months. The demarcation between various seasons is not clear as in temperate zones, especially in view of the monsoons i.e., heavy rains around July/August which are very important for the largely agrarian society in these parts. However, for the sake of this study, the seasons were divided as follows:[10]

Summer: May to July

Autumn/ Rainy/ Monsoon: August to October

Winter: November to January

Spring: February to April

## RESULTS

Socio-demographic profile of 13 follow up patients revealed that the mean age of patients (n=13) was 42.85 years (S.D, 8.87), 53.8% were males, 92.3% were married, majority (53.8%) hailed from rural areas and 61.5% were educated upto matriculation, 38.5% were involved in clerical work / shop owners / farming, followed by 23.1% housewives/ household work, 61.5% were from joint family type and 76.9% followed Hinduism as their religion.

Clinical profile: Recurrent depressive disorder (RDD) current episode moderate with/ without somatic syndrome (30.8%) was the commonest current diagnosis as per ICD-10 DCR; RDD (46.2%) was the commonest lifetime diagnosis as per ICD-10 DCR, followed by Bipolar affective disorder (15.4%).

The mean total duration of illness came out to be 13.08 years and the mean number of previous episodes was 4.27. In majority of patients, the most recent episode had onset in first half of the year (46.15 %). Pattern of season of onset revealed Summer (4), Spring (3), Winter (3), Fall (1) and Not known (2). Six patients had a positive family history of Affective Disorder and break up is as follows: 3 had family history of psychotic illness, followed by 2 with depression and 1 had anxiety disorder.

SPAQ: Mean SPAQ global severity in patients was 3.46 (S.D, 6.55; range 1-24) and description of response of patients in terms of month in which patients had best or worst response in terms of general feeling, gaining or losing weight, socializing, sleeping and eating is as given below:

Majority of patients did not find any particular month in which they felt best (76.9%), tended to gain most weight (84.6%), socialize most (92.3%), slept most (84.6%), ate most (84.6%), lost most weight (92.3%), socialized least (92.3%), felt worst (69.2%) and ate least (84.6%). The commonest months in which these changes were reported were April to June.

Majority of patients did not report change in mood or energy in cold weather (46.2%), hot weather (53.8%), humid weather (53.8%), sunny days (53.8%), dry days (53.8%), grey cloudy days (53.8%), long days (53.8%), high pollen count days (61.5%), foggy and smoggy days (61.5%) and short days (61.5%).

Season wise distribution of mean sleep hours is as follows: winter (6.46, S.D, 3.82), spring (6.23, S.D, 3.24), summer (5.85, S.D, 3.24) and fall (5.46, S.D, 3.60). Majority of patients (53.8%) did not show any weight fluctuations, change in food preferences (92.3%) or problem in experiencing changes with season (53.8%).

## DISCUSSION

It can be concluded that in follow up patients with a diagnosis of affective disorder the global seasonality score (GSS) was quite low (mean 3.46, S.D, 6.55; range 1-24) with majority of the cohort reporting only mild changes throughout the year. Majority of the cohort did not report any variation in mood, behaviour, sleep pattern and weight fluctuations. Therefore, it is difficult to relate seasonal changes to the processes underlying the development of affective disorder. Thus, manifestation of an affective disorder is not necessarily associated with high seasonality change.

Our findings do generate more speculation rather than clearing doubts regarding seasonality and SAD, at least in India. Research on SAD otherwise too has shown that seasonal variations in mood and behaviour is a continuous, dimensional variable ranging from no change to full blown SAD.

Our results also raise questions about the usefulness of the SPAQ to function as a measure of seasonal variation in mood and behaviour, which is in keeping with the previous longitudinal studies on seasonality. A recent review[1] highlighted the difficulties related to consistency of diagnosis of SAD, using SPAQ. These studies did not find stability of diagnosis and that nearly 50% of the subjects did not retain their regular pattern. In another longitudinal study,[15] it was found that the validity of SSI (SPAQ score index) between two consecutive years was good, but decreased as the time difference between tests increased. Also, there was no difference found between seasonality defined by DSM-III-R and SSI.

Limitation of this study is the relatively small sample size and the findings are discussed in context of the climatic conditions of North India. The cohort comprised patients with all types of affective disorders; not solely being restricted to major depression. There is need to carry out more studies on larger samples and across different seasonal zones.
